# From gains to liver pain: when exercise training goes too far

**DOI:** 10.20517/evcna.2025.36

**Published:** 2025-06-25

**Authors:** Benjamin I. Burke, John J. McCarthy, Ahmed Ismaeel

**Affiliations:** ^1^Department of Physiology, College of Medicine, University of Kentucky, Lexington, KY 40506, USA.; ^2^Center for Muscle Biology, College of Health Sciences, University of Kentucky, Lexington, KY 40506, USA.

**Keywords:** Extracellular vesicles, overtraining, liver fibrosis, skeletal muscle, inter-organ communication, exercise physiology

## Abstract

A recent study from Liu *et al.* described the role of skeletal muscle-derived extracellular vesicles in promoting liver fibrosis as an outcome of chronic overtraining in mice. Here, we highlight this work and discuss its implications within the fields of exercise physiology and inter-organ communication.

## MAIN TEXT

### Introduction

Due to increases in skeletal muscle metabolism and contraction during exercise, there has been a growing interest in understanding how the release of various factors from skeletal muscle tissue may influence other organs^[1-3]^. Recently, the release of skeletal muscle-derived extracellular vesicles (SMEVs) has emerged as a mechanism for inter-organ communication^[3-7]^. Previous research has suggested that SMEVs may contribute to the pleiotropic effect of exercise on whole-body health and exercise adaptations^[1,2,8-12]^. For example, exercise-stimulated release of SMEVs has been found to play a role in adipose tissue lipolysis and osteogenesis in bone. However, recent findings by Liu *et al.* reveal the unexpected negative effects of SMEVs on liver health in response to exercise overtraining^[13]^.

### Research summary

Liu *et al.* conducted an exercise training study where healthy participants were classified as either an “overtraining group” or “control group” based on their perceived exertion during a cycling training program^[13]^. Participants in the overtraining group had higher liver enzyme levels and liver fibrosis-related markers. To corroborate these findings, they subjected mice to 12 weeks of forced treadmill running, finding that overtraining, but not moderate exercise, led to liver fibrosis as determined by liver pro-fibrotic gene expression and histology. Mice with pre-existing liver conditions also had exacerbated outcomes with overtraining. These data suggest that overtraining can lead to liver damage in both humans and mice.

Mechanistically, the authors found overtraining caused an accumulation of lactate in skeletal muscle, which, upon release into circulation, induced liver fibrosis. Through a series of elegant rescue experiments, Liu *et al.* showed that overtraining-induced liver damage can be blunted via skeletal muscle-specific knockdown of lactate dehydrogenase (*Ldha*), the enzyme responsible for producing lactate^[13]^. C2C12 myotubes treated with lactate also released extracellular vesicles (EVs), which induced apoptosis in hepatocytes *in vitro*. Moreover, using skeletal muscle-specific labeling of EVs, the authors demonstrated that SMEVs are transferred to the liver *in vivo*. The authors then isolated SMEVs from moderately trained, overtrained, and *Ldha*-deficient overtrained mice, which were subsequently administered to healthy mice. While treatment with SMEVs from control overtrained mice induced liver fibrosis, mice receiving SMEVs from *Ldha*-deficient overtrained mice were protected from liver fibrosis.

To determine how lactate influences SMEVs to promote liver damage, Liu *et al.* performed proteomics on SMEVs isolated from control- and lactate-treated C2C12 myotubes, finding that F-box protein 2 (FBXO2) was enriched following lactate treatment^[13]^. The silencing of *Fbxo2* alleviated the pro-apoptotic effects elicited by SMEVs isolated from lactate-treated myotubes on hepatocytes. Through immunoprecipitation-mass spectrometry technology, the authors identified SH3 domain-containing 3 (SORBS3) as a key protein interacting with FBXO2. In response to lactate treatment, SORBS3 was shown to undergo lactylation, leading to its liquid-liquid phase separation and, subsequently, the selective sorting of the SORBS3-FBOX2 complex into SMEVs. To confirm these findings, the authors subjected skeletal muscle-specific SORBS3-deficient mice to overtraining and found a significant reduction in FBXO2^+^ SMEV release and attenuation of liver fibrosis. The skeletal muscle-specific knockdown of FBOX2 similarly reduced both FBXO2^+^ SMEV release and liver damage. Furthermore, treatment of wild-type mice with either FBXO2^-^ or FBXO2^+^ SMEVs demonstrated that only FBXO2^+^ SMEVs induced hepatic fibrosis.

Finally, the authors determined that these FBXO2^+^ SMEVs act by reducing the expression of myeloid cell leukemia-1 (MCL1), an anti-apoptotic factor and binding partner of FBXO2. By downregulating MCL1, FBXO2^+^ SMEVs led to increased apoptotic factors and subsequent apoptosis in hepatocytes. Conversely, overexpression of MCL1 in the liver protected against the pro-apoptotic effects of FBXO2^+^ SMEV treatment, further confirming the role of MCL1 in FBXO2^+^ SMEV-mediated hepatic fibrosis.

### Limitations and conclusions

There are some important limitations that should be taken into consideration when interpreting the findings of the study. First, any attempt to replicate human exercise in mice is imperfect. While murine models of exercise are necessary and admittedly useful for discovery^[14,15]^, their translatability is limited. Although overly strenuous exercise has been previously linked to liver damage in humans^[16,17]^, it remains to be determined whether the precise mechanisms presented in this study are conserved in humans. There is alternative cargo carried in SMEVs, such as microRNAs, which may influence hepatic metabolism and be implicated in liver health^[18]^, and therefore, the proposed mechanism may not be exclusive. It would be of great interest to know if athletes such as triathletes or cross-fit athletes, who are highly susceptible to overtraining given their high-volume training, show a higher incidence of liver damage. Alternatively, the proposed mechanism might not apply to highly trained athletes due to training-induced adaptations that provide some form of protection against liver damage. Although the authors show data suggesting that overtraining in humans may lead to liver damage, caution is necessary given how overtraining was assessed through indirect metrics (i.e., self-reported total metabolic equivalent, self-reported participant exertion) and that some markers used in the present study (i.e., AST) may be indicative of muscle injury rather than liver damage^[16]^. Therefore, it remains to be seen how effectively the mechanisms described in this paper can be corroborated in humans.

Through this work, Liu *et al.* establish a possible downside to exercise-stimulated inter-organ communication in the context of overtraining^[13]^. Notably, overtraining has previously been shown to result in other negative effects on skeletal muscle metabolism^[19]^. While recent studies have explored the positive effects SMEVs can have on other organs^[1,2,8-12]^, EVs were originally thought to be a mechanism of waste disposal^[20]^. Although strenuous exercise is largely beneficial to health and longevity and should be universally encouraged, the present study does well to remind us that, while EVs certainly mediate many important functions critical to health, we must consider and explore the potential disadvantages of tissue crosstalk.

## DECLARATIONS

### Copyright

© The Author(s) 2025.
